# Interactive Responses of Potato (*Solanum tuberosum* L.) Plants to Heat Stress and Infection With Potato Virus Y

**DOI:** 10.3389/fmicb.2018.02582

**Published:** 2018-10-30

**Authors:** Svetlana Makarova, Antonida Makhotenko, Nadezhda Spechenkova, Andrew J. Love, Natalia O. Kalinina, Michael Taliansky

**Affiliations:** ^1^Shemyakin-Ovchinnikov Institute of Bioorganic Chemistry of the Russian Academy of Sciences, Moscow, Russia; ^2^A.N. Belozersky Institute of Physico-Chemical Biology, Lomonosov Moscow State University, Moscow, Russia; ^3^The James Hutton Institute, Dundee, United Kingdom

**Keywords:** potato virus Y, heat stress, *S. tuberosum*, thermotolerance, antivirus defense

## Abstract

Potato (*Solanum tuberosum*) plants are exposed to diverse environmental stresses, which may modulate plant–pathogen interactions, and potentially cause further decreases in crop productivity. To provide new insights into interactive molecular responses to heat stress combined with virus infection in potato, we analyzed expression of genes encoding pathogenesis-related (PR) proteins [markers of salicylic acid (SA)-mediated plant defense] and heat shock proteins (HSPs), in two potato cultivars that differ in tolerance to elevated temperatures and in susceptibility to potato virus Y (PVY). In plants of cv. Chicago (thermosensitive and PVY-susceptible), increased temperature reduced *PR* gene expression and this correlated with enhancement of PVY infection (virus accumulation and symptom production). In contrast, with cv. Gala (thermotolerant and PVY resistant), which displayed a greater increase in *PR* gene expression in response to PVY infection, temperature affected neither PR transcript levels nor virus accumulation. *HSP* genes were induced by elevated temperature in both cultivars but to higher levels in the thermotolerant (Gala) cultivar. PVY infection did not alter expression of *HSP* genes in the Gala cultivar (possibly because of the low level of virus accumulation) but did induce expression of *HSP70* and *HSP90* in the susceptible cultivar (Chicago). These findings suggest that responses to heat stress and PVY infection in potato have some common underlying mechanisms, which may be integrated in a specific consolidated network that controls plant sensitivity to multiple stresses in a cultivar-specific manner. We also found that the SA pre-treatment subverted the sensitive combined (heat and PVY) stress phenotype in Chicago, implicating SA as a key component of such a regulatory network.

## Introduction

Potato (*Solanum tuberosum* L.), one of the most important non-grain food commodities in the world, is a cool-weather crop that has optimal growth at temperatures ranging between 14 and 22°C. Above these temperatures, potato yield is dramatically reduced (Van Dam et al., 1996), a consequence of biochemical and physiological perturbations in processes such as photosynthesis, tuberization and dormancy (Reynolds et al., 1990; Hancock et al., 2014). It is expected from currentclimate models that such heat stress effects on potato crops will become increasingly common, with potentially devastating impacts on potato production in many areas of the world (Li et al., 2013).

To cope with heat stress, plants have evolved a variety of mitigation strategies that promote thermotolerance (Bita and Gerats, 2013; Bakthisaran et al., 2015). For example, increased temperature may trigger remodeling of various signal transduction pathways which helps to confer thermotolerance by inducing changes in the structure of organelles, the cytoskeleton, and membrane morphology and fluidity (Bita and Gerats, 2013). In parallel with these changes, a family of HSP proteins (including HSP70, HSP90 and small HSPs like HSP20) become highly expressed. These HSP proteins greatly contribute to thermotolerance as they act to appropriately refold/stabilize and protect proteins and specific translation factors from high temperature inactivation (Bita and Gerats, 2013; Bakthisaran et al., 2015; McLoughlin et al., 2016). It has been suggested that the regulation and maintenance of thermotolerance in plants is multifaceted and a variety of plant hormonal components such as the salicylic acid (SA) pathway, have been implicated in these processes (Bita and Gerats, 2013). Thus, plant thermotolerance is a complex multigenic trait determined by combined expression of a number of genes and their products.

In nature, potato, *S. tuberosum*, like other plant species, are simultaneously exposed to various abiotic and biotic stresses. Among biotic stress factors are viruses, fungi, oomycetes, bacteria, nematodes, parasitic weeds and insects. Viruses represent a class of major plant pathogens which cause approximately half of all emerging plant disease outbreaks (Bernardo et al., 2018). Of significant concern for potato cultivation are potyviruses, such as potato virus Y (PVY). PVY is considered the most harmful virus affecting potato with wide epidemics in Europe and other continents from the 1980s onward (Scholthof et al., 2011). Losses of up to 45% of production have been estimated following primary infection, but the biggest losses are experienced when the crop is grown from PVY infected seed (secondary infection), where yield reductions of up to 85% have been reported, depending on the cultivar, virus strain combination and year^1^.

It is well established that environmental cues including heat can significantly affect plant–pathogen interactions, possibly *via* the modulation of host defense responses (Prasch and Sonnewald, 2013). A broad range of defense responses which can break down at high temperatures has been reported, particularly for incompatible interactions such as *R* (resistance) gene-mediated resistance, which is characterized by a rapid necrotization around the pathogen entry site that prevents further pathogen spread. For example, the resistance mediated by the tobacco *N* gene (class of *R* gene encoding an NB-LRR structure) against tobacco mosaic virus ceases at 28°C and above; a phenomenon thought to be due to temperature-sensitive conformational loss of function changes in the NB-LRR protein (Zhu et al., 2010). A similar effect was reported for resistance to *Pseudomonas syringae* in Arabidopsis plants, and therefore it is likely that various other resistance-related NB-LRR proteins may also lose activity at elevated temperatures (Zhu et al., 2010). A number of *R* genes conferring hypersensitive response (HR; *Ny*) related resistance or extreme resistance (ER; *Ry*) to PVY have been identified in potato species (*Solanum* spp.) (Solomon-Blackburn and Bradshaw, 2007). Most of these resistance genes, such as *Ny* found in *S. sparsipilum* and *S. sucrense*, and *Ny-1* in potato cultivar Rywal, confer resistance only at low temperatures (16–20°C). At higher temperatures (24–28°C) resistance does not develop, and PVY spreads systemically throughout the plant. In contrast, resistance to PVY^N^ expressed in *S. stoloniferum* (*Rysto*) and *S. chacoense* (*Rychc*) is effective at both low (16–20°C) and higher temperatures (above 24°C) (Bradshaw and Ramsay, 2005; Solomon-Blackburn and Bradshaw, 2007). In the latter case, the *R*-gene product seems to be temperature-resistant and does not lose its activity at elevated temperatures.

Compatible plant–virus interactions, a process typified by successful whole plant systemic virus spread from the initially infected tissues, is also influenced by heat stress. For example, high temperatures significantly enhanced the susceptibility of Arabidopsis to turnip mosaic virus (TuMV) (Prasch and Sonnewald, 2013). Similarly, tomato plants subjected to heat stress were more sensitive to tomato yellow leaf curl virus (TYLCV) and produced increased amounts of the virus. On the other hand, it was found that TYLCV infection attenuated the heat stress response of infected tobacco plants (Anfoka et al., 2016).

In both incompatible and compatible plant-virus interactions, key phytohormones such as SA, jasmonic acid (JA) and ethylene act as major signaling molecules in triggering plant defense mechanisms (Love et al., 2005; Lewsey et al., 2009; Vlot et al., 2009). However, their involvement and particular roles are dependent on virus-host combination and environmental conditions (Love et al., 2005; Lewsey et al., 2009; Vlot et al., 2009; Baebler et al., 2011; Kogovšek and Ravnikar, 2013). Experiments using *NahG* transgenic potato plants (cv. Désirée) which are deficient in accumulation of SA, clearly indicated that SA plays an important role in potato resistance to PVY (Baebler et al., 2011). The SA-mediated defense pathways are usually integrated with induction of a variety of pathogenesis-related (PR) proteins (Whitham et al., 2003). Some PR proteins display direct antibacterial and antifungal activity (Bowles, 1990), but there is no evidence that PR proteins have any activities associated with suppression of virus infections (Carr et al., 2018). However, as the activation of the PR protein expression is a hallmark of systemic acquired resistance, these proteins are commonly regarded as markers for SA-mediated antivirus defense (Love et al., 2005). Interestingly, SA is also involved in the regulation of HSP production and heat stress tolerance (Nazar et al., 2017).

Complementarily, HSPs (in particular HSP70 and HSP40) are known to be directly involved in virus replication (Hofius et al., 2007; Hafrén et al., 2010; Nagy and Pogany, 2010; Jungkunz et al., 2011; Mäkinen and Hafrén, 2014), and virus infections at early stages may modulate (facilitate or inhibit) synthesis of HSPs (Whitham et al., 2003). For example, the susceptible potato cv. Igor responded to PVY infection by increased production of HSPs, whereas in the resistant potato cv. Sante, HSP production was inhibited (Baebler et al., 2009).

Another type of plant antiviral defense response is RNA silencing. RNA silencing is a versatile and complex gene regulation and defense mechanism occurring in a broad range of eukaryotic organisms which mediates not only defense responses but also different endogenous regulatory processes involved in abiotic stress tolerance and growth and development (Eamens et al., 2008; Csorba et al., 2015). Remarkably, RNA silencing may also be dependent on temperature. Typically, RNA silencing-mediated defense is facilitated by rising temperatures, which may concomitantly reduce the development of virus diseases (Szittya et al., 2003; Chellappan et al., 2005; Tuttle et al., 2008). In contrast, as mentioned above in other plant-virus combinations, higher temperatures may lead to enhanced virus replication, resulting in more severe disease symptoms (Shams-Bakhsh et al., 2007; Prasch and Sonnewald, 2013; Anfoka et al., 2016; Obrępalska-Stęplowska et al., 2015). *In toto*, this suggests that particular components of the complex plant defense system may dominate in different virus–plant combinations and that they may be further differentially modulated by elevated temperatures. This scenario is rendered more complicated by the fact that RNA silencing (Lewsey et al., 2010; Zhou et al., 2014) and virus-encoded silencing suppressors are associated with phytohormone-mediated defense pathways (Love et al., 2012; Laird et al., 2013; Csorba et al., 2015), which can also in turn be regulated by temperatures (Shams-Bakhsh et al., 2007).

Collectively, all these observations strongly suggest that molecular pathways induced in responses to virus infections and heat stress can overlap and interact with each other, however, the mechanisms of such interactions are largely unknown. To shed more light on the interactive molecular responses to combined heat stress and virus infection in potato, comparative analysis of single and combined stress responses was carried out on two potato cultivars differing in tolerance to heat stress caused by elevated temperatures and susceptibility to PVY. We investigated whether and how potato resistance to PVY was affected by enhanced temperature, or whether and how PVY infection influenced heat stress responses. As a first step toward understanding the interplay between these responses, we analyzed the expression of genes encoding PR protein markers of the SA-mediated plant defense pathway and also heat shock proteins. Additionally, we explored the effect of external SA application on heat stress responses in the presence or absence of PVY and found that SA can subvert the susceptibility to heat and PVY in the sensitive potato cultivar.

## Materials and Methods

### Virus, Plant Material and Growth Conditions

Ordinary PVY strain O (PVYO; Gibson et al., 1990) hereinafter referred to as PVY, was maintained and propagated in *Nicotiana tabacum.* Potato (*S. tuberosum* L.) cultivars Gala and Chicago were grown and maintained by micro-propagation in stem node tissue culture. Two weeks after node segmentation, they were transferred to soil and after 1 month of growth the potato plants were used for heat treatment and PVY infection experiments. Plants were maintained for the duration of the experiments in controlled growth chambers (Pol-Eko-Aparatura, Poland) with a 16/8 h day/night photoperiod at a relative humidity of 60% with a light fluence of 250 μmol m^-2^ s^-1^.

### Electrolyte Leakage Assay

To assess thermotolerance by measuring cell membrane injury, an electrolyte leakage assay was carried out, essentially as described in Trapero-Mozos et al. (2018). Four 10-mm leaf disks each from a different leaf per plant were placed in test tubes containing 5 mL of distilled water. These were incubated in water baths at 22 and 47°C with continuous shaking for 5 h. After cooling to room temperature total conductivity was measured in samples using a conductivity meter. The extent of cell membrane injury was calculated by measuring the percentage of difference in conductivity between 47 and 22°C.

### Plant Inoculation, Heat Treatment and Application of Salicylic Acid

Fresh PVY^O^ infected *N. tabacum* plant material was ground in liquid nitrogen to a fine powder using a mortar and pestle, after which cold 125 mM potassium phosphate (K-P) buffer pH 7.5 was added at a ratio of 1:3 (w/v). The extract was centrifuged at 13000 *g* for 10 min at 4°Ñ and the supernatant was used for inoculation. For inoculation, two leaves of each plant were dusted with celite and rubbed with either 50 μL of virus inoculum or K-P buffer pH 7.5 (for mock inoculated controls). Inoculated leaves were then twice washed with water. Two days post inoculation (dpi) half of the plants were transferred to 28°C (moderate heat treatment); while the other half remained at 22°C. Leaf tissue samples from three mock- and virus-inoculated plants (two leaves per plant) were collected and pulled together at different time points [2, 3, and 8 dpi from inoculated leaves and 8, 14, and 21 dpi from upper non-inoculated (emerging, newly developed) leaves] and used for quantitative RT-PCR (qRT-PCR) analyses. For SA treatment experiments, plants were sprayed with either a control solution of 0.11% (v/v) ethanol or 1 mM SA dissolved in 0.11% (v/v) ethanol at 1 day prior to virus/mock inoculation and then daily for three consecutive days. All data are presented as mean values from six biological replicates (two from each of three experiments); each replicate was composed of samples from three plants pulled together (two leaves per plant).

### RNA Extraction and Real Time Quantitative RT-PCR (qRT-PCR)

Species-specific prefixes (St) are used in this section and figure legends to define mRNAs corresponding to the *S. tuberosum* genes: *StPR1-b, StPR2, StHSP90, StHSP70, StHSP20 (StHSP20-21), StCox* and *StEF-1α*. However, for simplicity, in the main body of the manuscript this “St” nomenclature is not used for *S. tuberosum* genes, proteins or mRNAs. Leaf tissue was ground into a fine powder under liquid nitrogen using a mortar and pestle, and RNA was extracted from the grindate using the ExtractRNA reagent (Evrogen, Russia) according to manufacturer recommendations. The purity of the RNA samples was determined by absorbance readings at 260/280 nm, and the RNA integrity was verified by electrophoresis in a 1% agarose 1xTBE gel stained with ethidium bromide. Residual DNA was removed by treating RNA with the RNase-free DNase I kit according to the manufacturer’s protocols (Invitrogen, Schwerte, Germany). Aliquots of 2 μg of DNase-treated RNA were reverse transcribed into cDNA using the SuperScriptTM First-Strand Synthesis System for RT-PCR (Invitrogen), in conjunction with either an oligo-dT primer (for host plant-specific mRNAs) or a PVY specific primer (see Supplementary Table S1). The primer pairs for SYBR green-based real-time PCR analysis of PVY^O^ RNA (primer pair was designed using PRIMER EXPRESS software), StPR-1b mRNA (Baebler et al., 2011); StPR-2 (β-1,3-glucanase, GluIII) mRNA (Kogovšek et al., 2010), StHSP70 mRNA (PGSC0003DMG400011197 – Potato Genomics Resource at Michigan State University; Gong et al., 2015), StHSP90 mRNA (Sołtys-Kalina et al., 2015), StHSP20-21 mRNA (as a member of the HSP20 group; Zhao et al., 2018) are listed in Supplementary Table S1. Primer concentrations giving the lowest threshold cycle (C_t_) value were utilized in RT-PCR and are also shown in Supplementary Table S1. Real-time RT-PCR was carried out in an ABI PRISM 7700 Sequence Detection System (Applied Biosystems, United States) on 10-fold dilutions of first-strand cDNA reaction mixes using the procedures described in the QuantiTect^TM^ SYBR^®^ Green PCR kit (Qiagen). All reactions were heated to 95°C for 15 min, followed by 40 cycles of 94°C for 15 s, 60°C for 30 s and 72°C for 30 s. All calculations and statistical analysis were performed as described in the ABI PRISM7700 Sequence Detection System User Bulletin #2 (Applied Biosystems, United States). The C_t_ value for PVY and each mRNA was normalized to mRNAs encoding cytochrome c oxidase subunit 1 (StCOX; Baebler et al., 2011) and StEF-1α (Nicot et al., 2005) respectively; primers are listed in Supplementary Table S1.

### Statistics

The data are presented as the mean ± SEM (standard error of the mean) of 6 biological replicates as described above and were analyzed using a two-tailed Student’s *t*-test (unpaired). All analyses were performed using GraphPadPrism software (Version 5.0). Differences were considered to be significant when the exact *P*-value was < 0.05. Correlations were tested using the Pearson correlation coefficient (r) at http://www.alcula.com/calculators/statistics/correlation-coefficient/ and the associated statistical test was used to evaluate whether the correlation was significant.

## Results

To establish the experimental system for studying the interaction between responses to heat stress induced by elevated temperatures and also PVY infection, we used two potato cultivars described as either resistant (cv. Gala)^2^ or sensitive (cv. Chicago)^3^ to PVY infection. Based on the symptoms at elevated temperatures observed in our preliminary screening experiments, we have also provisionally classified potato cv. Gala as a relatively thermotolerant cultivar, whereas potato cv. Chicago has been regarded as a cultivar with a relatively thermosensitive phenotype.

### Quantitative Evaluation of Thermotolerance in Potato Cultivars Gala and Chicago

**FIGURE 1:**
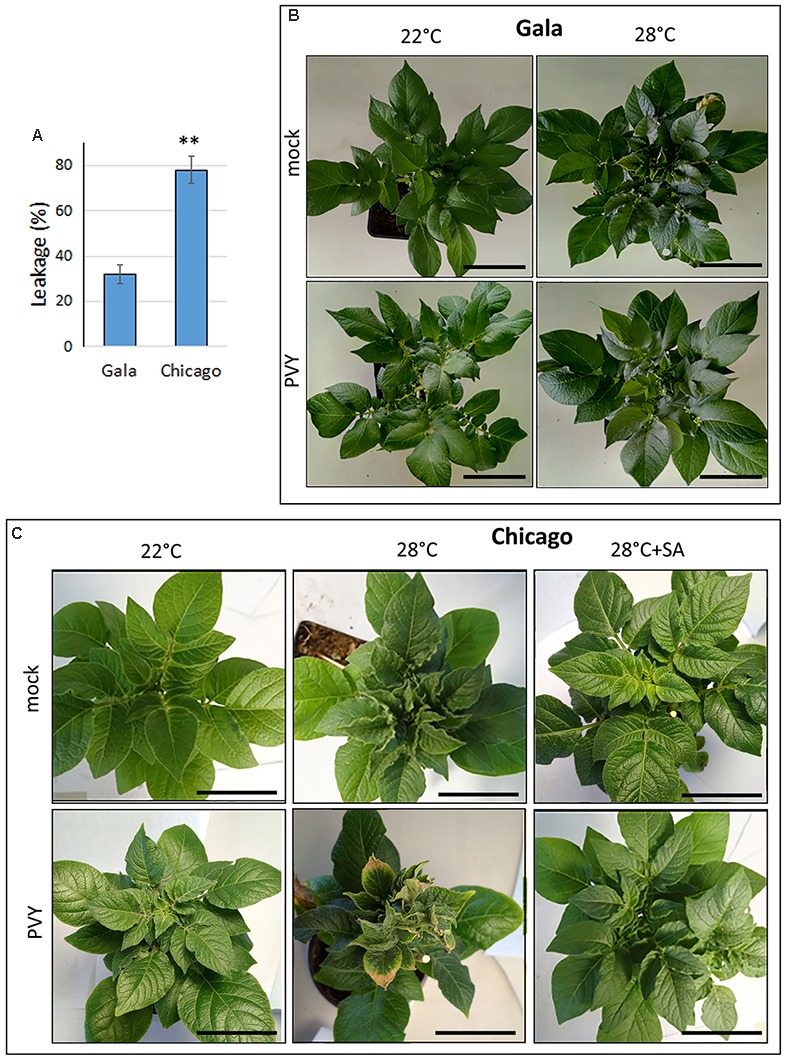
Thermotolerance and effect of elevated temperature on PVY infection in two potato cultivars: Gala and Chicago. **(A)** Thermotolerance was assessed by measuring electrolyte leakage (cell membrane injury) which was calculated by measuring the percentage difference in conductivity between 47 and 22°C. Typical patterns of symptom expression in mock- and PVY-inoculated Gala **(B,C)** Chicago plants grown at 22°C (normal) or 28°C (elevated) temperatures at 21 dpi. Right column in **(C)** displays symptoms of mock- or PVY-inoculated Chicago plants after treatment with salicylic acid (SA) 21 dpi. Scale bar, 10 cm. Typical pictures with lateral view of the plants are shown in Supplementary Figure S1.

To confirm these observations and provide quantitative evaluation of the thermotolerance, we determined how the cell membrane integrity changes in these cultivars in response to temperatures by measuring leakage of electrolytes (Ahn et al., 2004; Savić et al., 2012; Trapero-Mozos et al., 2018). In this work, potato leaf disks were subjected to high (47°C) and normal (22°C; control) temperatures for 5 h, and electrolyte leakage was calculated by measuring the percentage of difference in conductivity between the samples at 47 and 22°C. Potato cv. Gala displayed significantly lower electrolyte leakage (32%) than cv. Chicago (78%) at high temperature (Figure 1A), confirming that cv. Chicago is much more sensitive to heat than cv. Gala.

### Effect of Elevated Temperature on Symptom Expression and PVY Infection in Cultivars Gala and Chicago

To examine the impact of heat stress on the development of PVY infection in Gala and Chicago, plants grown under ambient conditions were inoculated with the virus and incubated at 22°C for 2 days prior to half of them being transferred to 28°C, while the other half remained at 22°C. The moderately increased temperature conditions (28°C) were chosen to mimic long-term mild heat stress that plants may be exposed to in nature, and to avoid excessively high temperatures which could rapidly kill the plants.

We did not observe any significant differences in the appearance of mock-inoculated cv. Gala plants grown at elevated (28°C) versus normal (22°C) temperature, except that their upper leaves seemed to be slightly more rigid and waxy at the higher temperature (Figure 1B). In contrast, mock-inoculated Chicago plants displayed some obvious visible symptoms such as curling of the outer edges of the top leaves after 8–10 days at 28°C, which was not observed in plants that remained at 22°C (Figure 1C). This confirms that sensitivity to moderately elevated temperature stress is greater for Chicago versus Gala; this is also consistent with the data presented above on the differences in thermotolerance between these cultivars under severe (47°C) heat stress (Figure 1A).

In juxtaposition to the largely asymptomatic plants of cv. Gala infected with PVY at both 22°C and 28°C, PVY-infected plants of cv. Chicago developed relatively mild systemic symptoms at 22°C which were strongly exacerbated by higher (28°C) temperature (Figure 1C). In this case symptoms appeared in the top non-inoculated leaves at 10 dpi in the form of leaf deformation, curling and crinkling followed by plant stunting, leaf growth retardation, browning leaf edges and lower leaf drop; thus significantly exceeding the severity of the phenotype in mock-inoculated plants kept at this temperature (Figure 1C and Supplementary Figure S1).

**FIGURE 2:**
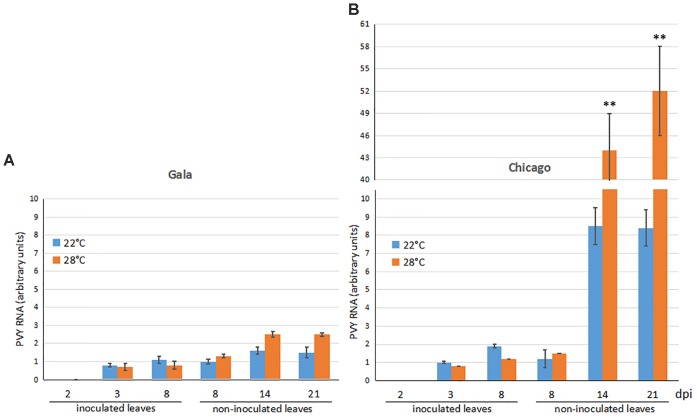
Accumulation of PVY RNA (measured using qRT-PCR) in inoculated and non-inoculated newly emerging systemically infected leaves of potato cultivars Gala **(A)** and Chicago **(B)** over a 2, 3, 8, 14, and 21 dpi time period at 22 or 28°C. Data points are the mean ± SEM; *n* = 6 from three independent experiments. ^∗^*P <* 0.05; ^∗∗^*P <* 0.01 (Student’s *t*-test).

PVY RNA accumulation was measured by qRT-PCR in inoculated and upper non-inoculated (emerging) leaves of cultivars Gala and Chicago infected by the virus. In inoculated leaves of both cultivars, patterns of PVY accumulation were essentially similar: PVY was detected by 3 dpi at low levels which remained relatively constant over time and were not significantly affected by temperature (Figures 2A,B). Moreover, in both cultivars PVY was able to spread systemically, invading upper leaves at 8 dpi (Figures 2A,B). These data suggest that the virus replication rates at the sites of inoculation as well as timing of systemic (long-distance) spread do not differ between both cultivars and were not affected by elevated temperature.

With regards to non-inoculated leaves, great differences in the rates of PVY accumulation were observed between Gala and Chicago. In Gala plants the PVY RNA levels in non-inoculated leaves did not significantly increase at 22°C and only very slightly increased at 28°C (less than 2-fold) after 8 dpi (Figure 2A). In contrast, in non-inoculated leaves of cv. Chicago, a significant rise in PVY RNA levels was detected in the upper leaves during the same time period at 22°C (up to seven-fold) and was further dramatically increased (a 30-fold increase) at 28°C (Figure 2B).

Collectively these data suggest that Gala exhibits a distinct type of resistance to PVY, whereby the virus, in spite of its ability to infect plants systemically, does not produce any apparent symptoms and only replicates at very low levels that do not increase over time in either inoculated or systemically infected leaves. Moreover, the type of resistance in this thermotolerant cultivar is not affected by elevated (28°C) temperature. In contrast, increased PVY RNA levels were found in systematically infected leaves of the thermosensitive cv. Chicago particularly at elevated temperature, which could suggest that increased temperature may compromise this cultivar’s defense responses against PVY.

### Differential Expression of Genes Associated With Defense Responses to Virus Infection at Normal and Elevated Temperature in Cultivars Gala and Chicago

To examine whether PVY infection triggers defense responses, we analyzed the gene expression of two PR-proteins which are traditional hallmarks of activation of the SA-mediated signaling pathway in potato; namely PR1-b (Hoegen et al., 2002; Baebler et al., 2011) and PR-2 (β-1,3-glucanase, GluIII) (Baebler et al., 2011).

**FIGURE 3:**
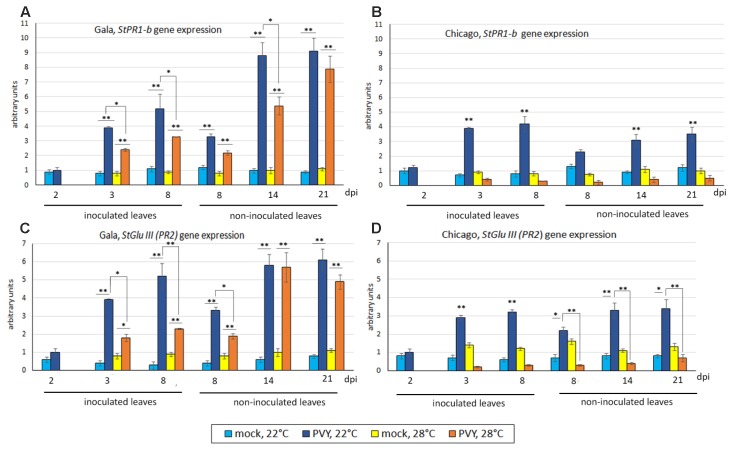
Expression level of PR protein genes: *StPR1-b* **(A,B)** and *StGluIII* (*PR2*) **(C,D)** (measured using qRT-PCR) in inoculated and non-inoculated newly emerging systemically infected leaves of potato cultivars Gala **(A,C)** and Chicago **(B,D)** at 22 or 28°C at 2, 3, 8, 14, and 21 dpi as shown. Data points are the mean ± SEM; *n* = 6 from three independent experiments. ^∗^*P <* 0.05; ^∗∗^*P <* 0.01 (Student’s *t*-test).

At 2 dpi under normal temperature (22°C), levels of expression of *PR1-b* and *GluIII* in PVY- inoculated leaves of Gala and Chicago were low and comparable to mock-inoculated leaves. However, from 3 dpi onward, significant and similar increases in the expression of both these genes were detected in virus- but not mock- inoculated leaves in both Gala and Chicago plants (Figures 3A–D). In upper (newly developed, emerging) non-inoculated leaves of PVY-infected Gala and Chicago plants, levels of PR1-b and GluIII transcripts were found to be elevated from 8 to 21 dpi, in comparison to the mock-inoculated controls (Figures 3A–D). However, such increases in levels of PR1-b and GluIII transcripts were significantly higher in the case of Gala versus Chicago at 14 and 21 dpi (*p* ≤ 0.01). Interestingly in these tissues, it was found that Chicago had higher rates of PVY accumulation compared with Gala; which is indicative of a negative correlation between expression of PR protein genes and virus accumulation (*r* = -0.89, *p* ≤ 0.01 for *PR1-b*; *r* = -0.78, *p* ≤ 0.01 for *GluIII*).

At elevated temperature (28°C), expression of *PR1-b* and *GluIII* in both PVY-inoculated (3–8 dpi) and systemically infected leaves (8–21 dpi) of Chicago was suppressed to similar or lower levels to those observed in mock-inoculated plants (Figures 3A–D). It was also observed that the down-regulation of the SA markers was coincident with enhanced systemic PVY infection in Chicago at elevated temperature (manifested in both symptom severity and virus accumulation as indicated in Figures 1C, 2B). Although some decrease in the levels of *PR1-b* and *GluIII* gene expression was also observed at elevated temperatures in PVY-inoculated and systemically infected upper leaves of Gala compared to normal (22°C) temperature, this effect was much less pronounced than in Chicago. Thus, significant levels of PR1-b and GluIII transcripts remained in PVY-inoculated and “systemically infected” leaves of Gala at both 22 and 28°C (Figures 3A–D) which is in good agreement with the resistance pattern of this cultivar to PVY infection at both normal and elevated temperatures.

In the absence of virus infection, elevations in temperature (28°C) in general did not affect the PR1-b and GluIII transcript levels in the leaves of either Gala or Chicago plants (Figures 3A–D).

Taken together these data suggest that elevated temperature can compromise PVY-triggered expression of SA markers in the sensitive cultivar Chicago. This suggests a down-regulation of the SA pathway which may significantly increase its susceptibility to PVY. In contrast, with the resistant cultivar Gala, the impact of temperature on resistance and suppression of the SA pathway was minimal.

### Differential Expression of Heat Shock Proteins in Response to Heat Stress and Virus Infection in Cultivars Gala and Chicago

To determine how PVY infection affects the moderate heat stress response of Gala and Chicago, we analyzed *HSP* genes belonging to three subfamilies: *HSP20*, *HSP70* and *HSP90*. It was previously shown that *HSP20* (*HSP20-21* used in this work) is significantly up-regulated in potato under heat stress (Zhao et al., 2018), whereas HSP70, in addition to its function in response to heat stress, plays several roles in virus infections (reviewed in Nagy and Pogany, 2010). The other marker we analyzed, HSP90, was shown to be essential for efficient plant responses to stress and heat tolerance (Anfoka et al., 2016).

**FIGURE 4:**
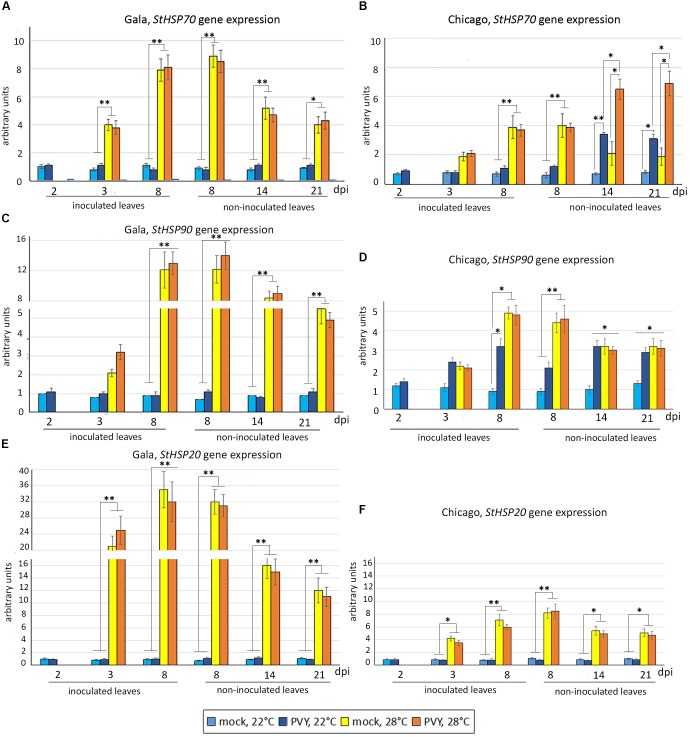
Expression level of *HSP* genes: *StHSP70* **(A,B)**, *StHSP90* **(C,D)** and *StHSP20* (*StHSP20*-21) **(E,F)** (measured using qRT-PCR) in inoculated and non-inoculated newly emerging systemically infected leaves of potato cultivars Gala **(A,C,E)** and Chicago **(B,D,F)** at 22°C or 28°C at 2, 3, 8, 14, and 21 dpi as shown. Data points are the mean ± SEM; *n* = 6 from three independent experiments. ^∗^*P <* 0.05; ^∗∗^*P <* 0.01 (Student’s *t*-test).

By 8 dpi in mock-inoculated Gala, the expression of all these genes was significantly enhanced by elevated temperature (28°C), although to different levels (highest for *HSP20*, and lowest for *HSP70*), after which time they declined (Figures 4A,C,E). Interestingly a similar profile was observed for PVY infected plants, indicating that PVY infection alone does not activate expression of any of these *HSP* genes at 22 or 28°C (Figures 4A,C,E).

With regards to Chicago (sensitive to heat and PVY), patterns of *HSP* gene expression in response to heat and PVY were more complex due to their differential expressions. The expression pattern of *HSP20* in Chicago was very similar to that observed in Gala; elevations in temperature triggered increased levels of expression of this gene, but these levels declined with time and were not influenced by PVY infection (Figures 4E,F). While the transcript levels of *HSP70* and *HSP90* essentially followed a similar trend in response to elevated temperature, PVY infection had an obvious effect on these inductions (Figures 4B,D). Although PVY infection increased *HSP70* levels in non-inoculated leaves at 22°C from 14 dpi onward, a synergistic increase in levels was observed during this time period in the presence of elevated temperature (Figure 4B). Unlike with *HSP70*, PVY strongly induced expression of *HSP90* in both inoculated leaves and non-inoculated leaves at 22°C, and moreover no synergistic increase in levels was observed in response to both PVY and temperature (Figures 4B,D). It is worth noting that expression levels of *HSP* genes (in particular, *HSP 20* and *HSP 90*) in Chicago were typically much lower than in Gala (Figures 4A,C,E versus Figures 4B,D,F) which correlates well with the different levels of sensitivity of these cultivars to heat stress.

In summary PVY had no notable effect on the heat stress responses in Gala, whereas in cultivar Chicago, PVY infection appears to induce HSP-related signaling pathways and further modulates HSP responses triggered by heat stress, implicating an interplay between molecular responses to PVY infection and heat stress.

### Salicylic Acid Subverts the Heat and PVY Sensitive Phenotype in Chicago Cultivar

Salicylic acid is widely accepted as a key modulator of defense mechanisms during various plant-virus interactions, including PVY infection in potato (Baebler et al., 2011; Zhou et al., 2014; Lee et al., 2016). It is also known that SA alleviates heat stress in a broad range of plant species (reviewed in Khan et al., 2015; Nazar et al., 2017) and mediates basal thermotolerance (Clarke et al., 2004).

**FIGURE 5:**
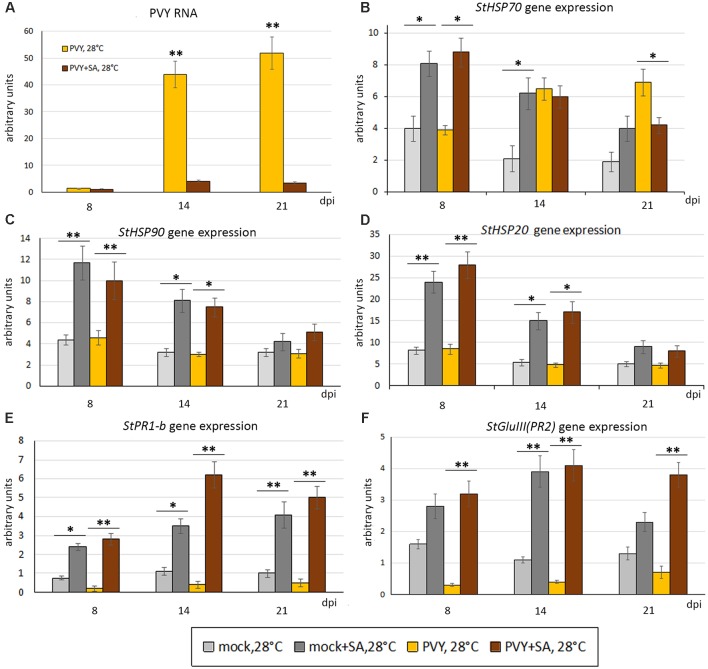
Effect of treatment with salicylic acid (SA) on accumulation of PVY RNA **(A)** and expression level of PR protein and *HSP* genes, *StHSP70* **(B)**, *StHSP90* **(C)**, *StHSP20* (*StHSP20*-21) **(D)** *StPR1-b* **(E)**, *StGluIII* (*PR2*) **(F)** (measured using qRT-PCR) in non-inoculated newly developed leaves of mock- and PVY-inoculated Chicago plants at 28°C at 8, 14, and 21 dpi as shown. Data points are the mean ± SEM; *n* = 6 from three independent experiments. ^∗^*P <* 0.05; ^∗∗^*P <* 0.01 (Student’s *t*-test).

To investigate whether SA confers resistance to combined stress induced by heat and PVY infection in Chicago (PVY and heat sensitive), a solution of 1mM SA or water [containing 0.11% (v/v) ethanol] was sprayed onto Chicago leaves 24 h prior to PVY or mock-inoculation, and then daily for 3 consecutive days. Following treatment with SA, the phenotypes that developed in mock- or PVY-inoculated plants at 28°C were significantly milder compared with the water-sprayed control plants (Figure 1C and Supplementary Figure S1). Consistently, accumulation of PVY RNA in systemically infected leaves of these plants were also dramatically decreased by SA treatment (Figure 5A).

To understand the role of SA in conferring resistance to heat and PVY infection in Chicago, we studied the dynamics of HSP and PR protein gene expression in response to heat stress and PVY infection, with or without SA treatment.

We confirmed that the SA pathway marker genes, *PR1-b* and *GluIII*, had strongly enhanced expression in both virus- and mock-infected Chicago plants at 28°C as a result of exogenous SA application (Figures 5E,F); which is consistent with the successful activation of the SA pathway and likely resultant reduction in PVY accumulation.

With regards to heat stress markers, we found that SA treatment induced significant increases in expression of *HSP90* and *HSP20* in 28°C grown mock-inoculated and virus-infected Chicago plants at 8 dpi, after which expression levels fell but were still higher than the water-treated controls (Figures 5C,D). In a similar manner, *HSP70* expression levels also increased after SA treatment in both mock- and PVY-infected plants at 8 dpi, however, after this time the expression pattern significantly fell to that of the water controls (14 dpi) or lower (21 dpi) (Figure 5B). These data on SA induced HSP transcript accumulation and thermotolerance in the Chicago cultivar confirms the role of the SA-mediated signaling pathway in modulating heat stress responses.

**FIGURE 6:**
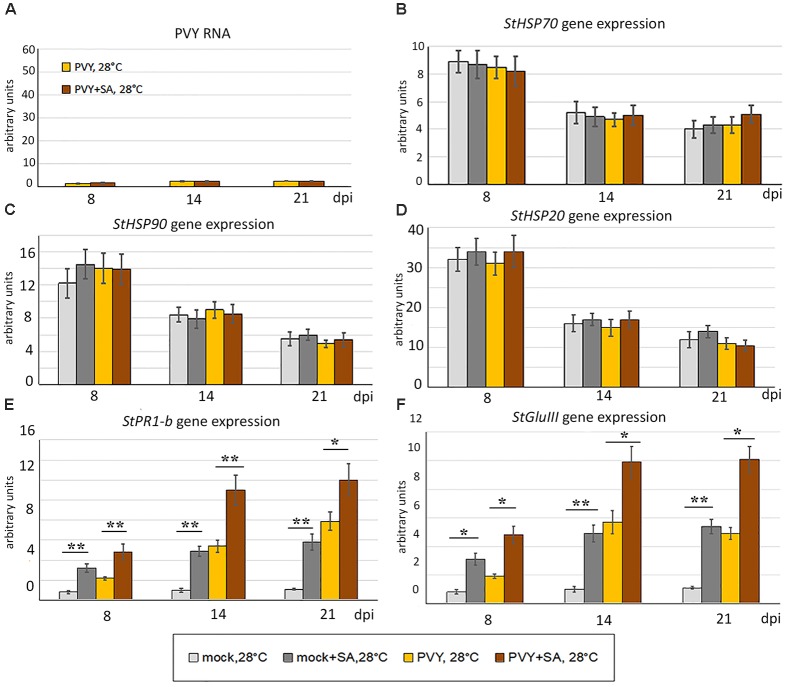
Effect of treatment with salicylic acid (SA) on accumulation of PVY RNA **(A)** and expression level of PR protein and *HSP* genes, *StHSP70* **(B)**, *StHSP90* **(C)**, *StHSP20* (*StHSP20*-21) **(D)** *StPR1-b* **(E)**, *StGluIII* (*PR2*) **(F)** (measured using qRT-PCR) in non-inoculated newly developed leaves of mock- and PVY-inoculated Gala plants at 28°C at 8, 14, and 21 dpi as shown. Data points are the mean ± SEM, *n* = 6 from three independent experiments. ^∗^*P <* 0.05; ^∗∗^*P <* 0.01 (Student’s *t*-test).

The phenotypes that developed in mock- or PVY-inoculated plants at 28°C in thermotolerant and virus-resistant Gala were not affected by SA treatment. Consistent with this, levels of PVY RNA accumulation were not influenced by SA in this cultivar (Figure 6). However, the expression of both *PR1-b* and *GluIII* was significantly increased by SA in Gala, suggesting further up regulation of SA-mediated signaling (Figure 6). In contrast, expression of all three *HSP* genes tested in this study (*HSP70*, *HSP90* and *HSP20*) was not significantly affected by SA treatment. These data suggest that internal mechanisms in Gala are sufficient to provide full defense against PVY under heat stress. These mechanisms (whether or not they are related to the SA-mediated pathway) remain to be elucidated.

## Discussion

Plants in nature are frequently exposed to diverse environmental and physiological stresses which may modulate plant–pathogen interactions, potentially further incurring significant reductions in crop productivity (Pandey et al., 2017). Consequently, there is a strong demand to study consolidated plant responses to combined abiotic and biotic stresses. One of the key environmental factors affecting plant-pathogen interactions is temperature elevations. Taking into account that temperature during the potato growing season is likely to increase, as indicated by climate change scenarios (Li et al., 2013), we studied the responses of potato plants to combined stress induced by elevated temperature and PVY, one of the most devastating potato pathogens.

It was previously described that cv Gala is resistant and cv Chicago is sensitive to PVY infection. However, in our experiments (at normal temperature) PVY resistance in Gala is not manifested as full abolition of the virus; but rather there is some detectable but low level of virus accumulation remaining in both inoculated and upper non-inoculated leaves, concurrent with impaired symptom development (Figures 1B, 2A). These data suggest that compared to Chicago, virus replication or/and cell-to-cell movement in Gala is strongly limited in upper systemically infected leaves, representing a previously described but mechanistically uncharacterized type of “resistance to PVY accumulation” (Barker and Dale, 2006; Palukaitis, 2012). Interestingly, in spite of the considerable contrast between the cultivars in systemic PVY invasion of non-inoculated leaves, they have essentially similar low levels of virus accumulation in the inoculated leaves (Figures 2A,B). The mechanisms underlying these dissimilarities in local and systemic infection with PVY in Gala and Chicago remain unclear, but a possibility is that PVY infection may differentially modulate the activation of defense-related genes in these hosts.

We also found that the two cultivars had different responses to increases in temperature; we determined that Gala was thermotolerant and Chicago was thermosensitive *via* the use of electrolyte leakage assays (Figure 1A). It was observed that the thermotolerant and PVY resistant Gala cultivar had only very slightly increased susceptibility to PVY upon temperature elevations, which was evident on upper non-inoculated (systemically infected) leaves (Figure 2A). In contrast, with the thermosensitive and PVY susceptible Chicago, temperature elevations resulted in far more severe symptoms in systemically infected (non-inoculated) leaves, with a great corresponding enhancement in the accumulation of PVY (Figures 1C, 2B). This observation corroborates the existence of strong links between responses to heat stress and PVY infection during the compatible virus–host interaction in cultivar Chicago.

The molecular linkages between temperature and its influence on virus infection have been previously well documented for incompatible plant–virus interactions. With incompatible responses, the appropriate combination of virus-specific elicitor and host *R* gene triggers rapid activation of defense responses in a temperature-dependent manner, with high temperatures being inhibitory for defense (Bradshaw and Ramsay, 2005; Barker and Dale, 2006; Solomon-Blackburn and Bradshaw, 2007; Zhu et al., 2010; Palukaitis, 2012). In contrast, with regards to compatible plant-virus interactions, the mechanisms of temperature-dependent responses are much less well studied (Shams-Bakhsh et al., 2007; Prasch and Sonnewald, 2013; Del Toro et al., 2015; Obrępalska-Stęplowska et al., 2015; Anfoka et al., 2016) and totally uncharacterised in potato. Given the role of RNA silencing in plant antiviral defense and its possible dependence on temperature (Szittya et al., 2003; Chellappan et al., 2005; Tuttle et al., 2008) it could be suggested that this pathway may be compromised by elevated temperatures in the temperature sensitive Chicago cultivar, leading to enhanced accumulation of PVY. To test this hypothesis, we analyzed the accumulation of PVY-specific small interfering RNAs (siRNAs), the main hallmark of RNA silencing (Eamens et al., 2008; Csorba et al., 2015). The levels of PVY-specific siRNAs were not reduced in response to elevated temperature in Chicago or Gala (Supplementary Material and Supplementary Figure S2), indicating that temperature mediated enhancement of PVY accumulation occurs independently of (at least the initial steps of) the RNA silencing pathway. This suggests that other mechanisms of interplay between heat stress and PVY infection are responsible for this phenomenon.

Therefore, to gain new insight into the underlying cross talk between responses to heat stress and compatible virus infections in potato, we followed the expression of genes encoding heat-stress regulated HSPs and defense-related PR proteins in PVY-infected Gala and Chicago.

An important finding of this work is the highly pronounced differential effect of elevated temperatures and PVY infection on the expression of two classes of *PR* genes, *PR-1b* and *PR-2* (*GluIII*) in Gala versus Chicago. Although PR proteins have not been shown to be directly involved in anti-viral defense, they are valuable markers of activation of the SA pathway which can mediate defense against viruses in plants, including potato (Love et al., 2005; Baebler et al., 2011). We found that infected cv. Chicago had substantially lower levels of activation of the SA pathway (as suggested by *PR* gene expression) but significantly higher levels of PVY accumulation compared with Gala; an inverse correlation suggestive of a role of the SA pathway in reducing virus invasion in the Gala cultivar. However, it should be noted that in some other susceptible potato cultivars, PR protein genes are upregulated to much higher levels (Baebler et al., 2011), indicating that perhaps SA-mediated signaling may not always limit virus infection, suggesting involvement of other defense pathways. Nevertheless, why PR protein genes are expressed to different levels in susceptible cultivars remains to be investigated.

Interestingly, the negative relationship between PR protein expression and virus accumulation we observed was in turn modulated by changes in temperature, whereby increased temperature in Chicago plants further reduced *PR* gene expression and this correlated with an increase in PVY accumulation (Figures 2B, 3B,D). In contrast, with Gala, which had higher *PR* gene expression in response to PVY, temperature did not affect PR transcript levels nor virus accumulation (Figures 2A, 3A,C).

The corollary of these results is that the expression of *PR* genes during compatible interactions in potato may be regulated by elevated temperature in a cultivar-specific manner. These data conform to a previously published report (Prasch and Sonnewald, 2013), which showed that in another plant-virus compatible combination (Arabidopsis – TuMV), *PR* genes are down-regulated under heat stress. Collectively these findings suggest that basal defense during some (but not all) compatible virus-plant interactions may be compromised by heat stress.

To further support this idea and examine the impact of PVY infection on heat stress responses, we presented data showing differential effects of virus infection on the expression of three different *HSP* genes (*HSP70* versus *HSP90* and *HSP20*) in Gala and Chicago.

First, as expected, mild heat stress (28°C) induced expression of all the tested *HSP* genes in mock-inoculated plants of both Gala and Chicago. However, all three *HSP* genes were typically expressed to a higher level in Gala than in Chicago, which is consistent with greater basal thermotolerance in Gala (Figure 4).

Secondly, in Chicago even at normal temperature, PVY is able to induce expression of two of the three tested *HSP* genes (*HSP90* - from 3 dpi onward and *HSP70* – at later stages of infection) whereas expression of *HSP20* remains unchanged. Interestingly, up-regulation of *HSP* gene expression in response to PVY infection was also previously reported in another susceptible cultivar, Igor (Baebler et al., 2009); however, this, like Chicago (*HSP70/HSP90*), was in contrast to Gala which had no observed activation of *HSP* genes induced by PVY (Figure 4). This suggests that the different *HSP* gene activations may be triggered not only by abiotic stress but also by virus infection, and that this may be further modulated in a cultivar specific manner.

Finally, at elevated temperature when *HSP* genes are activated by heat stress, PVY does not generally further modulate expression of *HSP* genes, with the exception of *HSP70* which is up-regulated in Chicago at later stages of infection (Figures 4B,D,F). This *HSP70* up regulation may be a consequence of high levels of virus replication in plants at this time, which may be consistent with direct involvement of HSP70 in virus replication (Hofius et al., 2007; Hafrén et al., 2010; Nagy and Pogany, 2010; Jungkunz et al., 2011).

Collectively, these data confirm that responses to PVY infection and abiotic (heat) stress may share some underlying mechanisms in certain cultivars, which is exemplified by activation of *HSP* expression by PVY in Chicago at normal temperature. However, a distinct gene expression program may be activated in response to combined stress as a result of integration of individual stress-responsive signaling cascades (Rizhsky et al., 2004).

It has been suggested that phytohormones form a central hub that links, integrates and re-programs multiple abiotic stress responses (Golldack et al., 2014). There is good reason to suggest that plant hormones may also play an important role in integrating and reprogramming responses to combined abiotic and biotic stress. Remarkably, SA has been shown to affect susceptibility of plants to various pathogens including viruses in both incompatible and compatible combinations and responses to abiotic stresses (drought, low and high temperatures, UV and high salinity) (Love et al., 2005; Lewsey et al., 2009; Vlot et al., 2009; Zhou et al., 2014; López-Gresa et al., 2016; Dempsey and Klessig, 2017). SA also plays a pivotal role in various signaling pathways related to plant growth and development and basic biological processes, such as respiration, photosynthesis and the Krebs cycle (reviewed in Dempsey and Klessig, 2017). However, the mechanisms underlying these multiple effects are still largely uncharacterised, but it is clear that they involve coordinated action of SA and other plant regulators, such as JA, ethylene and many others. Several lines of research also suggest that SA-mediated pathways may be inter-related with RNA silencing (Ding et al., 2001; Alamillo et al., 2006; Jovel et al., 2011; González et al., 2012; Lee et al., 2016).

Basal levels of SA in potato plants are rather high and their increase in response to virus or fungal infection is usually moderate (Kogovšek et al., 2010), which can complicate its regulatory role. On the other hand, the direct role of SA in responses to PVY infection has been previously demonstrated by Baebler et al. (2011). Moreover, as a signaling molecule SA has been shown to induce thermotolerance in potato (Larkindale and Knight, 2002; Khan and Khan, 2013).

In agreement, here we have shown that in response to PVY infection, both Gala and Chicago potato cultivars demonstrate increases in SA-induced *PR* gene expression with a negative correlation between expression of PR proteins and virus accumulation (Figure 5). Moreover, we present evidence that pre-treatment of Chicago plants with SA mediates resistance to the combined stresses of heat and PVY, which coincides with increases in *PR* and *HSP* gene expression (Figure 5). However, this effect of SA is apparently not related to RNA silencing because SA did not increase accumulation of PVY-specific siRNAs (Supplementary Material and Supplementary Figure S2).

However, we are well aware that the mechanisms controlling susceptibility or resistance of plants to multiple stresses cannot rely on a single regulatory component. It is evident that integrated regulatory networks shape a specific program of responses to a particular combination of stresses. Therefore, elucidation of such regulatory networks is absolutely required for effective breeding of sustainable plant resistance to viruses in the face of climate change; identification of such regulatory networks will in future be elaborated using high throughout (omics) approaches.

## Author Contributions

MT and NK conceived and designed the study. SM, AM, and NS performed the experiments. MT, NK, and AL analyzed the data. MT and AL wrote the manuscript.

## Conflict of Interest Statement

The authors declare that the research was conducted in the absence of any commercial or financial relationships that could be construed as a potential conflict of interest.

## Footnotes

1 https://www.fera.co.uk/media/wysiwyg/crop_health/Factsheets/AphidTransmittedVirusesPotato.pdf

2 http://www.binst.be

3 http://varieties.ahdb.org.uk
