# Supplementary material for: Interactive Responses of Potato (Solanum tuberosum L.) Plants to Heat Stress and Infection With Potato Virus Y
Source: Front Microbiol. 2018 Oct 30;9:2582. doi: 10.3389/fmicb.2018.02582 (PMC6218853; doi:10.3389/fmicb.2018.02582)
Supplement: Supplementary file 1 [file Data_Sheet_1.docx]

**SUPPLEMENTARY MATERIAL**

**Interactive Responses of Potato (*Solanum tuberosum* L.) Plants to Heat Stress and Infection with Potato Virus** Y

***Svetlana Makarova^1,2†^, Antonida Makhotenko^1,2†^, Nadezhda Spechenkova^1^, Andrew J. Love^3^, Natalia O. Kalinina^1,2^ and Michael Taliansky^1,3*^***

^1^Shemyakin-Ovchinnikov Institute of Bioorganic Chemistry of the Russian Academy of Sciences, Moscow, Russia

^2^Lomonosov Moscow State University, Moscow, Russia

^3^The James Hutton Institute, Dundee, United Kingdom

**Supplemental Table S1. Primers used for quantitative RT PCR**

| **Primer** | **5’-3’ sequence** | **Reference** | **Primer**  **concentration (nM)** | **E (%)** |
| --- | --- | --- | --- | --- |
| PVY^O^-F  PVY^O^-R | TATGATGGATTTGGCGACCACTTGT  TAAACTAGGCAGCTCTGCATCATG | This work  [KC017747](https://www.ncbi.nlm.nih.gov/nucleotide/KC017747.1?report=genbank&log$=nucltop&blast_rank=76&RID=SZK1TVTM01R)  GeneBank | 400 | 95.7 |
| StPR-1b-F  StPR-1b-R | GTATGAATAATTCCACGTACCATATGTTC  GTGGAAACAAGAAGATGCAATACTTAGT | Baebeler et al., 2011 | 350 | 100 |
| StPR-2-F  StPR-2-R | CCCTGGAGTTGTTGTAAATGATAATG  ATGCTACATACTCGGCCCTTGA | Kogovšek et al., 2010 | 350 | 93.8 |
| StHSP90-F  StHSP90-R | GTTCCCTTGCTTTTTGAGACCG  GGGAACTCCAATGCAGGCGTG | Sołtys-Kalina  et al., 2015 | 400 | 94.3 |
| StHSP70-F  StHSP70-R | CGCGGTCGTAACGGTTCCAGC  CTTCGCCCTCTCACACGCGGTC | Gong et al, 2015 | 250 | 95.5 |
| StHSP20-21-F  StHSP20-21-R | AGAACGATACTTGGCATCGG  AATGGACTTGACATCAGGCT | Zhao et al., 2018 | 350 | 96.1 |
| StCOX-F  StCOX-R | GGTCGGACATACCTGAAAC  CCAAAAGTATGAAAAGCTGGAG | Baebeler et al., 2011 | 350 | 97.3 |
| StEF-1α-F  StEF-1α-R | CTTGACGCTCTTGACCAGATT  GAAGACGGAGGGGTTTGTCT | Nicot et al., 2005 | 350 | 98.7 |

Full references are provided in the main text. Primer concentrations giving the lowest threshold cycle (C_t_) value were utilized in RT-PCR and are listed in the Table. E, efficiency of PCR amplification as calculated by CFX Manager Software.

**
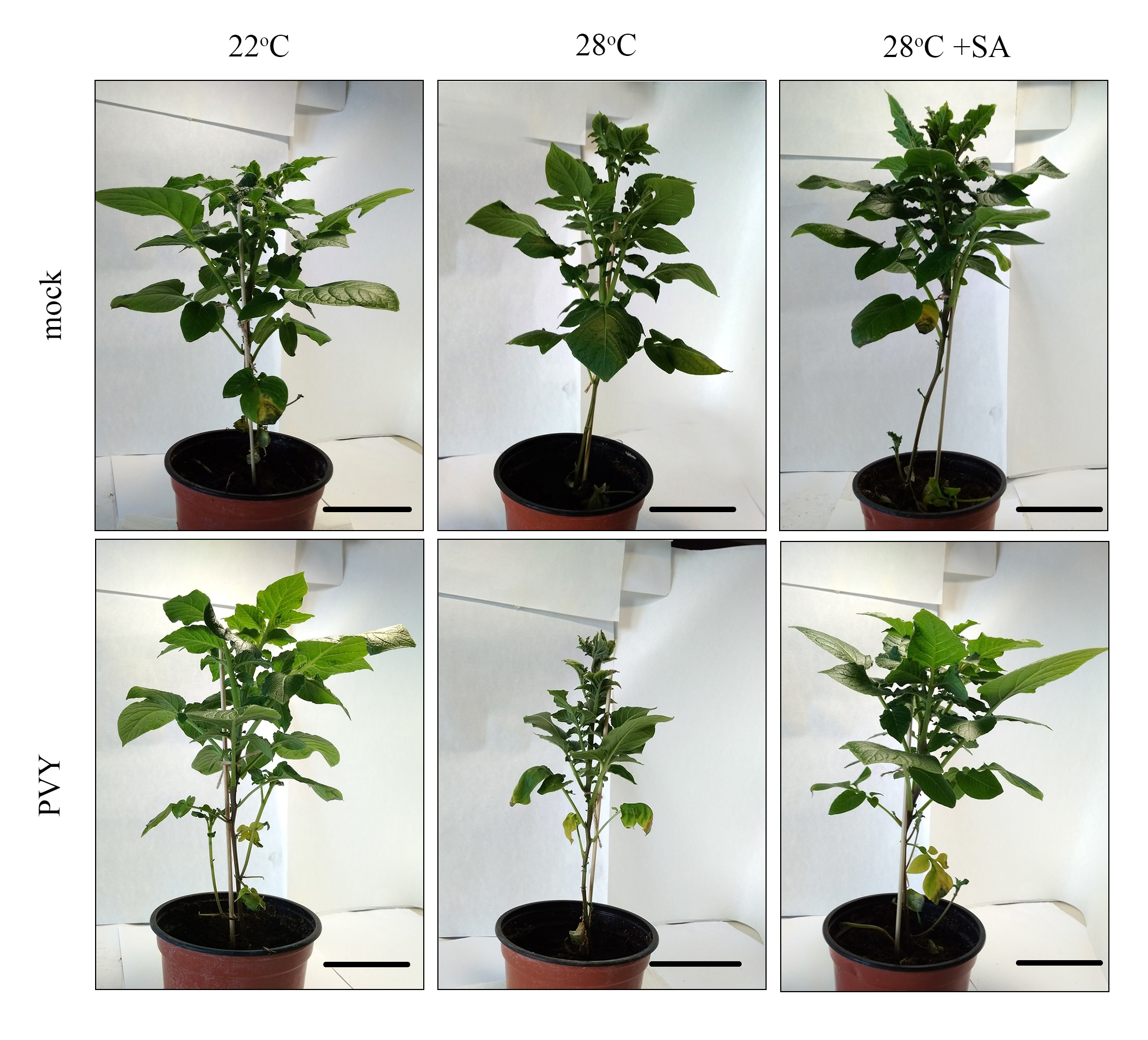
**

**Figure S1.** Typical symptoms in mock- and PVY-inoculated Chicago plants grown at 22°C (normal) or 28°C (elevated) temperatures at 21 dpi (lateral views). Right column displays symptoms of mock- or PVY-inoculated plants after treatment with salicylic acid (SA) 21 dpi. Scale bar, 10 cm.


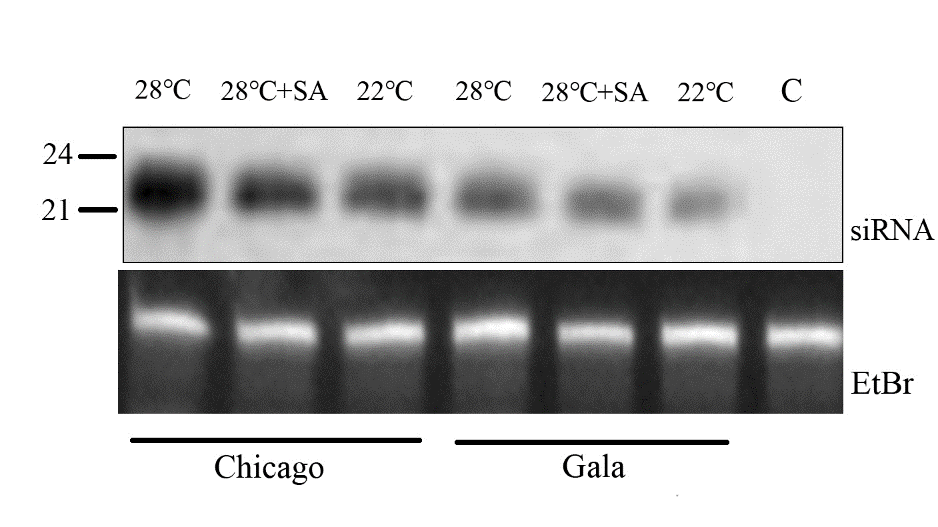


**Figure S2.** Detection (RNA gel blot analysis) of PVY-specific siRNAs in newly developed (emerging) leaves of PVY-infected Chicago and Gala plants grown at 22°C or 28°C and treated or untreated with SA at 14 dpi (as indicated). c - control Chicago mock-inoculated plants grown at 22°C. Ethidium bromide (EtBr) stained 5S rRNA is shown as loading control (which is abundant in the low molecular weight RNA population). Positions of 24- and 21-nt size markers are indicated.

The levels of PVY specific siRNAs were not reduced at elevated temperature in Chicago or in Gala. This suggests that significant increase in PVY RNA accumulation in Chicago at elevated temperatures (**Figure 2**) is not caused by (at least initial steps of) RNA silencing leading to the cleavage of viral dsRNAs into siRNAs. RNA silencing also does not appear to be an essential factor determining resistance of Gala plants to PVY since siRNA levels in Gala are generally lower than in virus-susceptible Chicago plants. Effect of SA increasing resistance of Chicago to PVY also presumably does not relate to RNA silencing because SA did not increase accumulation of PVY-specific siRNAs.

**Materials and Methods.** Total RNA was extracted from 1 g of leaf tissue using TRI REAGENT (Sigma) according to the manufacturer’s recommendations. Low-molecular weight (LMW) RNAs were resolved by electrophoresis in polyacrylamide/7M urea gels and then electroblotted onto Hybond N membrane and UV cross-linked using a StrataLinker (Stratagene). [^32^P]-labelled RNA probes corresponding to 5′ terminal region of PVY RNA were generated using the mMESSAGE mMACHINE T7 kit (Ambion Inc) with a random-priming DNA labelling system (Invitrogen). As a loading control, equal fractions of each sample were resolved on a 1% agarose gel and stained with ethidium bromide.
